# Β-Glucanase-driven changes in β-glucan structure and metabolism throughout Hull-less barley dough fermentation

**DOI:** 10.1016/j.fochx.2026.103809

**Published:** 2026-04-08

**Authors:** Ziyuan Guo, Xin Wang, Xiaoqing Deng, Luyu Fan, Qun Shen, Xiang Li, Xiaomeng Wu

**Affiliations:** a College of Culinary and Food Science Engineering, Sichuan Tourism University, 610100 Chengdu, China; b College of Food Science and Engineering, Shanxi Agricultural University, 030801 Jinzhong, China; c College of Food Science and Nutritional Engineering, National Engineering Research Center for Fruit and Vegetable Processing, Key Laboratory of Fruits and Vegetables Processing, Ministry of Agriculture, Engineering Research Centre for Fruits and Vegetables Processing, Ministry of Education, China Agricultural University, Beijing, China

**Keywords:** Hull-less barley, β-Glucan, Β-Glucanase, Structural characterization, Untargeted metabolomics

## Abstract

Hull-less barley is an important staple crop in plateau regions; however, its elevated β-glucan (BG) content often compromises dough quality. In this study, we investigated the mechanisms underlying β-glucanase (BGS)-mediated enhancement of hull-less barley dough quality by integrating structural characterization and untargeted metabolomics. BGS hydrolyzed BG glycosidic bonds, reducing its molecular weight from 6.577 × 10^5^ to 7.247 × 10^4^ Da and median psize (D₅₀) from 895.67 to 321.67 nm, thereby disrupting hydrogen bonding and lowering viscosity. Untargeted metabolomic analysis identified 768 metabolites, with BGS treatment significantly upregulating the levels of organic acids, lipid-like molecules, and organic oxygen compounds. D₅₀ exhibited significant negative correlations with amino acids associated with the umami taste, including glutamate and aspartate. These findings suggest that BGS-mediated BG depolymerization enhances substrate accessibility for enzymes and yeast; provides oligosaccharides as carbon sources; and promotes protein hydrolysis, lipid oxidation, flavor formation, and yeast fermentation.

## 1 Introduction

Whole grains are staple foods consumed globally, supplying a substantial portion of the energy required by the human body. They primarily comprise starch (70–72%), along with protein (7–15%), lipids (1–2%), water, and other substances (Terens et al., 2017). “Whole grains” refer to cereals that retain bran, germ, and endosperm, whereas refined grains are processed to remove the bran and germ. Thus, whole grains offer more dietary fiber, minerals, vitamins, and other beneficial phytochemicals than their refined counterparts. Significant inverse associations have been reported between the consumption of whole grains, bran, and dietary fiber and the risk of cardiovascular disease, suggesting that dietary fiber in whole grains may contribute substantially to cardiovascular protection (Barrett et al., 2019). Dietary fiber, often referred to as the “seventh nutrient” essential to humans, plays a crucial role in promoting health by exerting physical effects and regulating metabolism (Shen et al., 2012), thereby supporting intestinal function and reducing the risk of metabolic diseases.

Hull-less barley (*Hordeum vulgare* L. var. *nudum* Hook. f.), a major cereal crop in China's Qinghai–Tibet Plateau, is recognized for its substantial nutritional profile and functional properties. β-glucan (BG), the primary bioactive constituent of hull-less barley, is a polysaccharide composed of β-pyranosyl units connected by β-(1 → 3) and (1 → 4) glycosidic linkages. Its concentration ranges from 2.89% to 6.11% (Sammalisto et al., 2024), notably surpassing that in wheat, corn, and common barley varieties. BG confers multiple physiological benefits, including cholesterol-lowering effects, blood glucose regulation, immune system support, and improved intestinal health (Yao, Li, Qin, Ju & Wang, 2023).

Despite these advantages, the elevated dietary fiber content poses processing and sensory challenges. As a water-soluble dietary fiber, BG demonstrates high water-holding capacity and gel-forming ability (Mohebbi et al., 2019); these properties can interfere with gluten network formation during dough preparation. Additionally, high-molecular-weight BG competes with gluten proteins through physical dilution, whereas microgels formed at low temperatures infiltrate the gluten network during heating, leading to diminished structural integrity and compromised quality of fermented products. Steamed bread made with hull-less barley often exhibits reduced specific volume, firmer texture, sticky mouthfeel, and accelerated staling—factors that restrict its broader adoption and commercialization (Yang et al., 2024).

Microbial fermentation is a mild bioprocess that preserves bioactive constituents and converts macromolecules into high-activity, low-molecular-weight compounds, improving product quality. β-Glucanase (BGS), an enzyme that specifically hydrolyzes mixed-linkage (1 → 3) (1 → 4)-β-D-glucan (BG), has been widely utilized in the food industry. For instance, BGS reduces wort viscosity to improve filtration efficiency in beer brewing (Moreno Ravelo et al., 2024) and significantly enhances dough performance when incorporated into high–oat content products (Qian et al., 2022). Thus, it plays an essential role in processing cereal-based foods with elevated BG levels. Our previous research revealed that, in the Kunlun-14 variety, the addition of 0.1% BGS reduced BG content from 3.29 ± 0.28 to 1.96 ± 0.11 (Guo et al., 2026). Despite its widespread use, research to date has primarily focused on the macroscopic quality parameters of BGS-treated products; comprehensive investigations addressing structural modifications and related metabolomic mechanisms remain limited. Nevertheless, recent advancements in interdisciplinary analytical technologies have created new opportunities for detailed investigation of structure–function relationships in food components.

In the current study, we aimed to clarify the mechanisms by which BGS influences the quality of hull-less barley steamed bread at the molecular level. Specifically, we integrated multidimensional structural characterization with untargeted metabolomic analyses to evaluate the effects of different BGS supplementation levels (0.00%, 0.02%, 0.06%, and 0.10%) on the structural properties of BG and the metabolomic profile of hull-less barley dough, using Zangqing2000 (ZQ2000) as the raw material. The results of this study provide a theoretical foundation and technical support for developing high-quality barley-based food products. Furthermore, this work advances the understanding of the mechanistic role of BGS in hull-less barley processing, providing novel perspectives for precision processing and quality improvement in whole-grain food products.

## 2 Materials and methods

### 2.1 Materials

A hull-less barley cultivar, ZQ2000 (total starch: 64.60%, BG: 6.12%, amylose: 18.83%, and protein: 11.8%), along with a wheat cultivar (*Triticum aestivum* L.), Zhongkemai 138, were used in this study. Highly fermented active dry yeast was obtained from Angel Yeast Co., Ltd. (Henan, China), and sugar was supplied by Zhengzhou Rongping Food Co., Ltd. (Henan, China). BGS (1.3 × 10^5^ U/g) was obtained from Ningxia Xiasheng Biotechnology Co., Ltd. (Ningxia, China). All other chemical reagents were of analytical grade. Subsequently, the barley grains were milled into whole-grain flour (with bran retained) using SCIND CT410 device (FOSS Analytical, Denmark). All flour samples were sieved through a 100-mesh screen before subsequent analyses and stored at −4 °C until use.

### 2.2 Experimental groups

Four experimental groups were established as follows: the control group received a ZQ2000-based blend with 0% BGS (ZHB00); for the enzyme-treated groups, BGS was incorporated into the ZQ2000-based blends at concentrations of 0.02% (ZHB02), 0.06% (ZHB06), and 0.10% (ZHB10) (*w*/w, based on the total dry weight of the mixed flour). This concentration range was selected based on relevant literature concerning cereal processing and preliminary experiments (Yang et al., 2024).

### 2.3 Preparation of the hull-less barley dough

Dough was prepared by mixing hull-less barley whole-meal flour and wheat flour in a 7:3 ratio, along with yeast (*Saccharomyces cerevisiae*), sugar, wheat gluten, and baking powder. BGS was added at varying concentrations (0.00% [ZHB00], 0.02% [ZHB02], 0.06% [ZHB06], and 0.10% [ZHB10]) by replacing an equivalent portion of wheat flour. The dough-making process included ingredient incorporation (60 rpm for 3 min, followed by 100 rpm for 7 min), shaping, and fermentation (38 °C for 45 min at 85% relative humidity).

### 2.4 BG extraction

Dough BG was extracted using a hot-water extraction protocol adapted from Li et al. (2023). First, freeze-dried hull-less barley dough was pulverized and sieved through a 100-mesh screen. Samples (8 g) were suspended in aqueous ethanol (75% *v*/v) at a solid-to-solvent ratio of 1:8, then heated in an 80 °C water bath for 2 h. Subsequently, the residues were dried at 55 °C. The dried samples were rehydrated with distilled water (1,8, *w*/*v*), adjusted to pH 8.0, and extracted in a water bath for 2 h. Once cooled, the extracts were centrifuged at 6000 rpm for 10 min. The supernatant was adjusted to pH 6.4 and treated sequentially with 0.5 g thermostable α-amylase (4000 U) at 90 °C for 40 min, followed by 12 mL of pancreatin solution (2%, m/v) at 37 °C for 30 min. Enzymatic reactions were terminated by boiling the mixture for 20 min. The pH was adjusted to 4.5, and the solution was stored at 4 °C for 12 h. BG was precipitated with four volumes of 100% ethanol, redissolved in distilled water, and dialyzed (MWCO 8000–14,000 Da, 25 °C) for 72 h. After dialysis, the retentate was mixed with four volumes of ethanol, filtered, and the resulting precipitate was freeze-dried for 10 h before being ground into powder for storage.

### 2.5 Characterization of BG extracts

#### 2.5.1 Molecular weight

The molecular weight (Mw) and polydispersity index (PDI) of BG were measured using high-performance size exclusion chromatography, following the procedures described by Bai et al. (2019) and Wang et al. (2021), using DAWN HELEOS 8+ system equipped with Optilab T-rEX refractive-index detector and DAWN HELEOS II multi-angle laser light-scattering detector (Wyatt Technology, Santa Barbara, CA, USA). Separation was performed on TSKgel GMPWxl column (7.8 mm × 300 mm, Tosoh, Japan) with 0.1 M NaNO₃ as the mobile phase at a flow rate of 0.5 mL/min.

To prepare the sample, approximately 50 mg of BG was dissolved in 5 mL of the mobile phase. After complete dissolution and homogenization, the sample was left at 25 °C overnight and passed through a 0.45 μm filter (Millipore, Bedford, MA, USA) to obtain 2 mL of filtrate for analysis.

During testing, the chromatographic columns and detectors were maintained at 45 °C. All data were collected and processed using Astra 6.1 software (Wyatt Technology Corp.). A standard curve was established using dextran standards ranging from 1.0 to 670 kDa.

#### 2.5.2 Fourier transform infrared (FT-IR) and X-ray diffraction (XRD) analysis

The spectra of BG were analyzed using an FT-IR spectrophotometer (Thermo Fisher Scientific, CA, USA) operating within the range of 4000–400 cm^−1^ at a resolution of 4 cm^−1^. Approximately 5 mg of BG was finely ground with 200 mg of KBr and pelletized into a 1 mm thick disk.

XRD measurements were conducted on a D8 ADVANCE X-ray diffractometer (Bruker, Germany) equipped with Cu–Kα radiation, a standard ceramic sealed tube, and a LYNXEYE XE-T detector. The instrument was operated at 40 kV and 40 mA, scanning from 5° to 65° (2θ) at 6° per min. Diffraction patterns were subsequently processed using JADE 6.5 software (Materials Data Inc., Livermore, CA, USA).

#### 2.5.3 Analysis of thermal properties

The thermal stability of BG was assessed using a differential scanning calorimeter (NETZSCH STA 449 F5/F3, Netzsch, Germany). Approximately 10 mg of sample was placed in an aluminum pan (Φ 8 × 2.1 mm; Netzsch, Germany) and measured with an empty pan serving as the reference. The thermal program involved heating from 25 °C to 600 °C at 10 °C/min under a nitrogen flow of 100 mL/min. Data acquisition and analysis were performed using proprietary STARe software.

#### 2.5.4 Psize distribution analysis

The psize distributions of BG were determined using Malvern Mastersizer 3000 (Malvern Panalytical, Malvern, United Kingdom). The measurements included D_10_, D_50_, and D_90_ values, with D_50_ representing the median psize. Dispersion was evaluated according to the following Eq. (1):

(1) $$\mathrm{Dispersion}=\frac{D90-D10}{D50}$$

#### 2.5.5 Rheology analysis

The rheological properties were analyzed according to the method described by Yu et al. (2021), with slight modifications. For sample preparation, 0.3 g of BG was dissolved in water under continuous stirring in an 80 °C water bath. Measurements were performed by loading 3 mL of the prepared solution between 50 mm parallel plates with a 1 mm gap. Viscosity profiles were obtained at 25 °C, over a shear rate range of 0.1–1000 s^−1^, and frequency-scan measurements were performed using a rotational rheometer (MCR92, Anton Paar, Austria).

### 2.6 Metabolite analysis

#### 2.6.1 Sample preparation and extraction

Non-volatile flavor compounds were obtained from dough samples according to the methodology of Yan et al. (2019), with slight modifications. Dough samples were flash-frozen in liquid nitrogen, pre-frozen at −20 °C for 24 h, vacuum-lyophilized at −40 °C for 24 h, and cryogenically ground under liquid nitrogen protection. Samples (80 mg) were homogenized in 1 mL of extraction solvent (water: acetonitrile: isopropanol in equal parts) using cryogenic grinding for 1 min, followed by 30 min of ultrasonic extraction at 5 °C. After centrifugation at 12,000 rpm and 4 °C for 10 min, the supernatant was filtered and chilled at −20 °C for 1 h to precipitate proteins. Following centrifugation (12,000 rpm, 15 min, 4 °C), the supernatant was vacuum-dried and reconstituted in 200 μL of 30% acetonitrile. Prior to ultra-performance liquid chromatography–tandem mass spectrometry (UPLC–MS/MS), a final centrifugation step was performed (12,000 rpm, 15 min, 4 °C).

#### 2.6.2 UPLC–MS/MS

Metabolomic profiling of hull-less barley dough supplemented with varying levels of BGS was conducted using UPLC (Vanquish Horizon system, Thermo Scientific) coupled with tandem mass spectrometry (Q-Exactive HF-X, Thermo Scientific), following established protocols. Chromatographic separation was performed using ACQUITY UPLC HSS T3 column (100 mm × 2.1 mm, 1.8 μm, Waters Corporation, Milford, MA, USA) maintained at 40 °C. Sample injection volume was set at 2 μL, with a mobile phase flow rate of 0.3 mL/min. Mobile phase A comprised ultrapure water containing 0.1% formic acid, whereas mobile phase B consisted of acetonitrile with 0.1% formic acid. The gradient elution program was as follows: 100% A (0–1.0 min), linear transition to 5% A (1.0–12.0 min), maintained at 5% A (12.0–13.0 min), returned to initial conditions (13.0–13.1 min), and re-equilibrated (13.1–17.0 min). The column effluent was introduced into the mass spectrometer via electrospray ionization, and data were acquired in positive and negative ionization modes over a mass-to-charge ration (*m*/*z*) range of 70–1050. Total ion chromatograms were generated by summing the intensities of all detected ions within each mass spectrum.

#### 2.6.3 Data preprocessing and annotation

The raw LC–MS data were processed using Progenesis QI (Waters Corporation), which facilitated baseline filtering, peak detection, integration, retention time correction, and peak alignment. This systematic workflow generated a comprehensive data matrix featuring retention times, m/z ratios, and peak intensities, adhering to the 80% prevalence rule. Sample mass spectrometry peak response intensities were normalized using the sum normalization method to yield a final data matrix for subsequent analyses. Additionally, mass spectral data were cross-referenced with the Human Metabolome Database (HMDB) (http://www.hmdb.ca/) and Metlin database (https://metlin.scripps.edu/) to identify the detected metabolites.

### 2.7 Statistical analysis

All experiments were performed in triplicate, and results are presented as mean ± standard deviation (*n* = 3). The normality and homogeneity of variances were verified using the Shapiro-Wilk test and Levene's test, respectively. Statistical significance was determined via one-way analysis of variance followed by Tukey-Krammer multiple comparison in SPSS 22.0, with *P* < 0.05 considered significant. Data visualization was performed with Origin 2017 (OriginLab, Northampton, MA, USA) and the Metware Cloud platform (https://cloud.metware.cn/).

Original UPLC–MS/MS data were processed and analyzed via Progenesis QI software (Waters Corporation). Mass spectral information was matched to public metabolite databases (http://www.hmdb.ca and https://metlin.scripps.edu). Principal component analysis (PCA) and partial least squares discriminant analysis (PLS-DA) were performed using SIMCA 14.1 (Umetrics, Wilmington, CA, USA). The Kyoto Encyclopedia of Genes and Genomes (KEGG) database (https://www.kegg.jp) was used to identify metabolic pathways associated with differentially metabolized volatile compounds in hull-less barley flour.

## 3 Results and discussion

### 3.1 Mw analysis

**Fig. 1:**
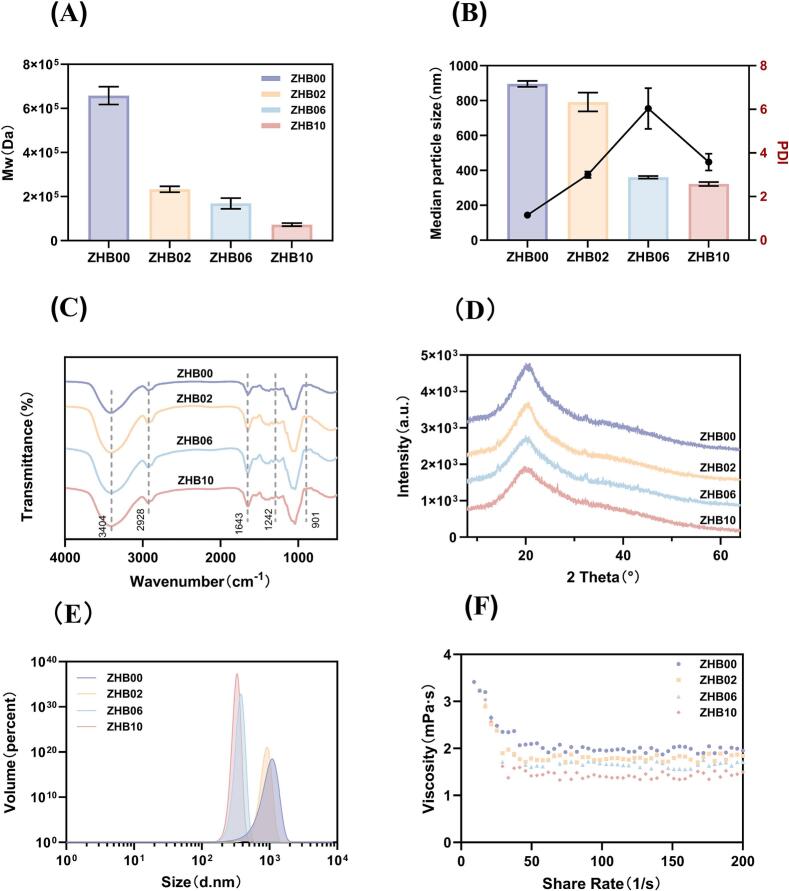
[cid:3e176dd1$1$19d6325db43$Coremail$dengxiaoqing0$163.com].

As shown in Fig. 1 A, the Mw of BG decreased with increasing BGS supplementation. Notably, ZHB10 had the lowest Mw of 7.247 × 10^4^ Da, indicating that higher enzyme concentrations promoted more substantial degradation. This differs from previously reported Mw values of hull-less barley BG, including 9.1 × 10^5^ Da documented by Kim and Kim (2017) and ranges of 2.2 × 10^5^–2.5 × 10^6^ and 8.2 × 10^5^–4.7 × 10^6^ Da reported by Storsley et al. (2003) using various methods.

The PDI displayed a nonlinear trend characterized by an initial increase followed by a subsequent decrease, ranging from 1.152 to 6.037 (Fig. 1B). ZHB00 had a PDI closest to 1, which suggests a relatively uniform BG distribution. As BGS concentration increased, the PDI increased sharply, peaking at 6.037 in ZHB06. Within the BGS range of 0.00–0.06%, the enzyme preferentially targeted BG chain segments with low steric hindrance, resulting in a heterogeneous system with undegraded long chains, randomly cleaved medium-length fragments, and fully degraded short-chain oligosaccharides. This heterogeneity resulted in an extremely broad Mw distribution, reflected in a notably high PDI. For ZHB10, the PDI exhibited a downward trend, suggesting enhanced degradation efficiency—abundant enzymes simultaneously acted on most BG chains, generating more uniform lower-molecular-weight BG fragments and thus reducing the PDI (Kupetz et al., 2016).

### 3.2 FT-IR spectra and XRD pattern analysis

BG samples exhibited generally consistent FT-IR spectral profiles in the 4000–400 cm^−1^ range (Fig. 1C), reflecting their uniform fundamental skeletal structures (Li et al., 2022). All samples displayed a broad absorption band near 1640 cm^−1^, attributed to O—H bending and C=O stretching (Gao et al., 2012; Kang et al., 2011), whereas the 2928 cm^−1^ band corresponded to C—H stretching vibrations (Hematian et al., 2017). The pronounced broadening at 3408 cm^−1^ likely resulted from enhanced intermolecular hydrogen bonding associated with the degradation of high-molecular-weight components (Hu et al., 2025), indicating inter- and intramolecular hydrogen bonding within polysaccharide networks (Guo et al., 2019). The 1200–800 cm^−1^ region serves as the fingerprint region for BG, with the emergence of double peaks near 1034 cm^−1^ potentially reflecting proportional variations in (1 → 3) and (1 → 4) glycosidic linkages across different BGS treatments, modifying C–O–C functional group vibrations. The absorption peak at 901 cm^−1^ confirmed the presence of glucopyranose residues, characteristic of the β-configuration (Yang & Zhang, 2009). Furthermore, FT-IR analysis identified a peak at 845 cm^−1^ corresponding to α-type glycosidic bond vibrations, attributed to residual starch impurities in the BG extracts, consistent with previous studies on barley BG extraction (Zhang et al., 2026). An absorption at 928 cm^−1^ was also observed, indicating predominant β-type glycosidic linkages (Qian et al., 2015).

All samples consistently exhibited structural features characteristic of polysaccharides, with molecular architectures comprising pyranose ring units linked by β-glycosidic bonds. Notable changes were detected in the C–O–C glycosidic bond stretching vibrations (1180–930 cm^−1^), likely attributable to reductions in Mw and modifications of β-glycosidic linkages following BGS-mediated degradation. Other absorption peaks remained largely unchanged after BGS treatment compared with those in the ZHB00 group, indicating that BGS supplementation did not alter the fundamental backbone structure of BG molecules.

The broad peak at 2θ = 20°, observed across all samples, consistent with Bai et al. (2021), reflects the coexistence of relatively ordered crystalline regions and amorphous domains formed during extraction (Ma et al., 2020) (Fig. 1D). The ZHB00 group demonstrated the broadest diffraction profile near 20°, suggesting the formation of gel-network structures via hydrogen bonding and other interactions among BG molecular chains, albeit with limited long-range ordering typical of amorphous configurations (Zhao et al., 2022). Increasing BGS supplementation led to decreased diffraction peak intensity, reduced integrated areas, and peak broadening. This likely resulted from the disruption of intermolecular hydrogen bonds and van der Waals forces within the crystalline regions by BGS, generating diverse crystal forms and polymorphs. The ZHB10 group exhibited pronounced alterations in its diffraction pattern, characterized by a considerable reduction in peak intensity at 20° and a notable broadening of the peak profile. This pattern indicates extensive hydrolysis of BG under high BGS supplementation, leading to degradation of long chains into oligosaccharides and short-chain fragments. Such substantial degradation profoundly disrupts the ordered hydrogen-bonded network among BG molecules, decreasing structural regularity and enhancing amorphous characteristics. The resultant shortened and disordered molecular segments exhibited reduced X-ray scattering capacity, resulting in markedly diminished diffraction intensity. However, the primary diffraction peak positions remained largely consistent across all samples, suggesting that although BGS affected the molecular chain arrangement, it did not alter the fundamental unit structure of the residual crystalline domains.

### 3.3 Analysis of thermal properties

Evaluation of the thermodynamic properties of hull-less barley dough revealed the highest denaturation peak temperature (Tp) and enthalpy change (ΔH) in the ZHB00 sample, reaching 88.29 °C and 2.21 J/g, respectively (Table 1). With increasing BGS supplementation, both Tp and ΔH progressively decreased, with the largest reduction observed in ZHB10 samples—Tp decreased by 5.36 °C and ΔH by 0.22 J/g compared with that in ZHB00. This suggests that BG modified by BGS is more susceptible to molecular structural changes or degradation during heating, which also affects cross-linking within gluten molecules and leads to depolymerization of the gluten network. In ZHB00, BG molecules exhibited strong intermolecular hydrogen bonding, requiring greater energy input to disrupt these interactions upon heating, resulting in a comparatively higher ΔH. Given the crucial role of hydrogen bonds in polysaccharide structural stability, ZHB00 samples exhibited superior thermal stability. Notably, compared to the ZHB06, ZHB10 showed slight increases in Tp, onset temperature (T0), conclusion temperature (Tc), and ΔH. This finding suggests that higher levels of BGS supplementation may induce local rearrangement of short-chain BG molecules or formation of new, less stable fragments, without fully restoring the original state of the control group.

**Table 1:** Thermal properties of samples with different β-glucanase (BGS) supplementation.

Samples	To (°C)	Tp (°C)	Tc (°C)	ΔH (mJ/mg)
ZHB00	86.18 ± 3.18^a^	88.29 ± 3.80^a^	89.78 ± 4.39^a^	2.21 ± 0.17^a^
ZHB02	82.15 ± 3.44^a^	84.05 ± 2.73^a^	85.71 ± 2.62^b^	2.08 ± 0.11^a^
ZHB06	79.68 ± 2.45^a^	81.40 ± 2.51^b^	83.25 ± 1.87^b^	1.93 ± 0.10^a^
ZHB10	80.82 ± 0.61^a^	82.49 ± 0.75^b^	84.03 ± 0.66^b^	1.99 ± 0.21^a^

Note: Data are expressed as the mean value of three replications ± standard deviation. Mean values with different letters within the same columns differ significantly (*P* < 0.05). Differential scanning calorimetry (DSC) thermal enthalpy determination was based on total solids. To: onset temperature of the peak (DSC); Tp: peak temperature of the peak (DSC); Tc: conclusion temperature of the peak (DSC); ΔH: melting enthalpy of the peak (DSC). ZHB00, ZHB02, ZHB06, and ZHB10 represent samples with 0.00%, 0.02%, 0.06%, and 0.10% BGS supplementation, respectively.

### 3.4 Psize analysis

The conformational properties of BG are typically represented by random coils, single helices, and multi-helical structures, which are influenced by intermolecular forces, temperature, and solvent conditions. Psize distribution provides insights into the dimensional attributes of BG molecules (Ma et al., 2022). Increasing BGS supplementation resulted in a progressive leftward shift in the distribution curve peaks (Fig. 1E), indicating a gradual reduction in psize and confirming the effective hydrolysis of BG by BGS. The highest D_50_ value (895.67 ± 17.16 nm) was recorded for ZHB00 samples, whereas D_50_ values decreased to 791.67 ± 54.08, 360.67 ± 7.64, and 321.67 ± 11.37 nm for ZHB02, ZHB06, and ZHB10, respectively. This phenomenon can be attributed to enzymatic degradation of BG by BGS into low-molecular-weight oligosaccharides and glucose monomers, resulting in a more concentrated particle-size distribution than the original profile (Taniguchi et al., 2022). Additionally, the narrowing of peak widths in the distribution curves with increasing BGS levels reflects the gradual homogenization of psize, consistent with the nonlinear trend in PDI variations (Gong et al., 2024).

### 3.5 Analysis of rheological properties

As the shear rate increased from 0 to 200 s^−1^, the apparent viscosity of all samples decreased (Fig. 1F). Each sample exhibited classic non-Newtonian, pseudoplastic characteristics. This behavior was likely due to disrupted intermolecular interactions at high shear rates, resulting in increased entropy. With higher shear rates, molecular chains increasingly align in the direction of flow and become less entangled. This alignment reduces fluid resistance, leading to a considerable decrease in viscosity. At sufficiently high shear rates, the molecular orientation approaches its limit, and viscosity stabilizes with minimal further variation (Diana et al., 2019).

With increasing BGS supplementation, the flow curves systematically shifted downward. At identical shear rates, the apparent viscosities of the enzyme-treated groups were significantly lower than that of the control group. This directly demonstrates that BGS disrupts the three-dimensional network sustained by hydrogen bonding and hydrophobic interactions through polymer chain cleavage, thereby reducing intermolecular friction, fluid resistance, and overall system viscosity. Higher enzyme supplementation leads to more extensive hydrolysis, which degrades macromolecules into smaller fragments and further decreases apparent viscosity. These findings highlight the potential of BGS as a flour improver capable of counteracting the high viscosity induced by BG and improving dough flexibility and processing performance.

### 3.6 Metabolomics analysis

#### 3.6.1 Analysis of metabolites

**Fig. 2:**
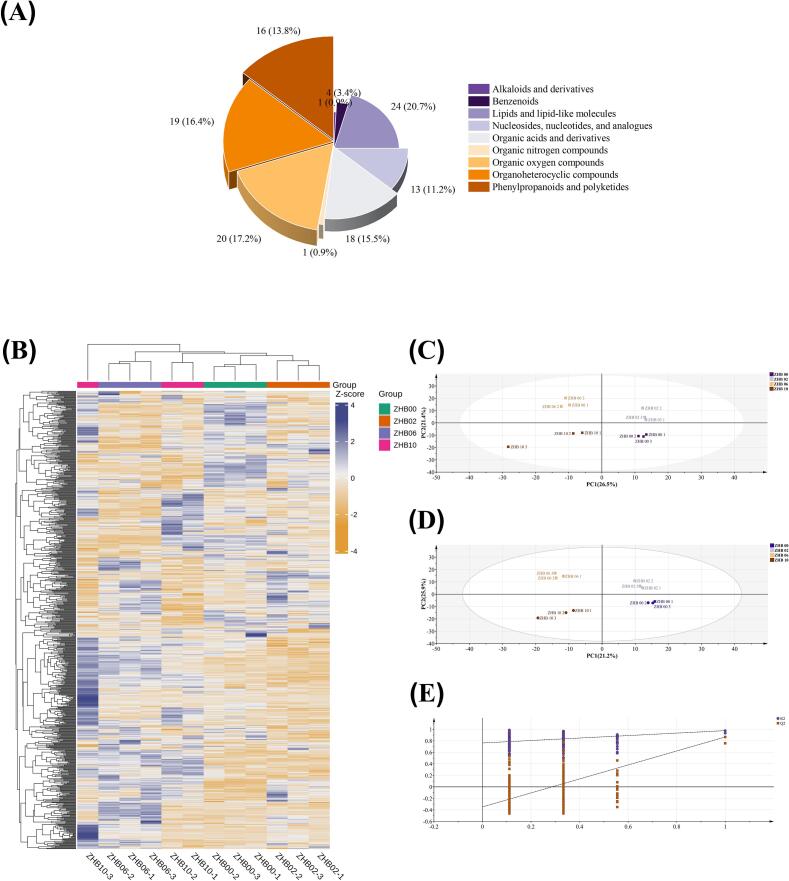
[cid:713f7d1d$2$19d6325db43$Coremail$dengxiaoqing0$163.com].

To comprehensively evaluate how BGS impacts metabolite profiles in hull-less barley dough, four sample types (ZHB00, ZHB02, ZHB06, and ZHB10) were selected for non-targeted metabolomics analysis using UPLC–MS/MS with a custom-built database. A total of 12 metabolite classes were detected (Fig. 2A), including organic acids and derivatives (45.31%), lipids and lipid-like molecules (11.72%), phenylpropanoids and polyketides (9.24%), organoheterocyclic compounds (5.34%), nucleosides, nucleotides, and analogues (4.56%), organic oxygen compounds (4.3%), benzenoids (2.60%), organic nitrogen compounds (0.65%), alkaloids and derivatives (0.39%), among others (Fig. 2B).

PCA plots revealed distinct sample separation, highlighting strong intragroup reproducibility and metabolic profile stability. The PC1 accounted for 49.57% of the variance, whereas PC2 accounted for 13.24% (Fig. 2C). Compared with PCA, PLS-DA provided greater resolution of metabolic differences related to BGS supplementation, as evidenced by clearer separation based on predefined classifications (Chen et al., 2009). Evaluation metrics for the model—R2X, R2Y, and Q2—were 0.713, 0.939, and 0.835, respectively (Fig. 2D), indicating higher predictive power. Statistical significance was further validated by permutation testing (Fig. 2E).

#### 3.6.2 Analysis of differential metabolites

**Fig. 3:**
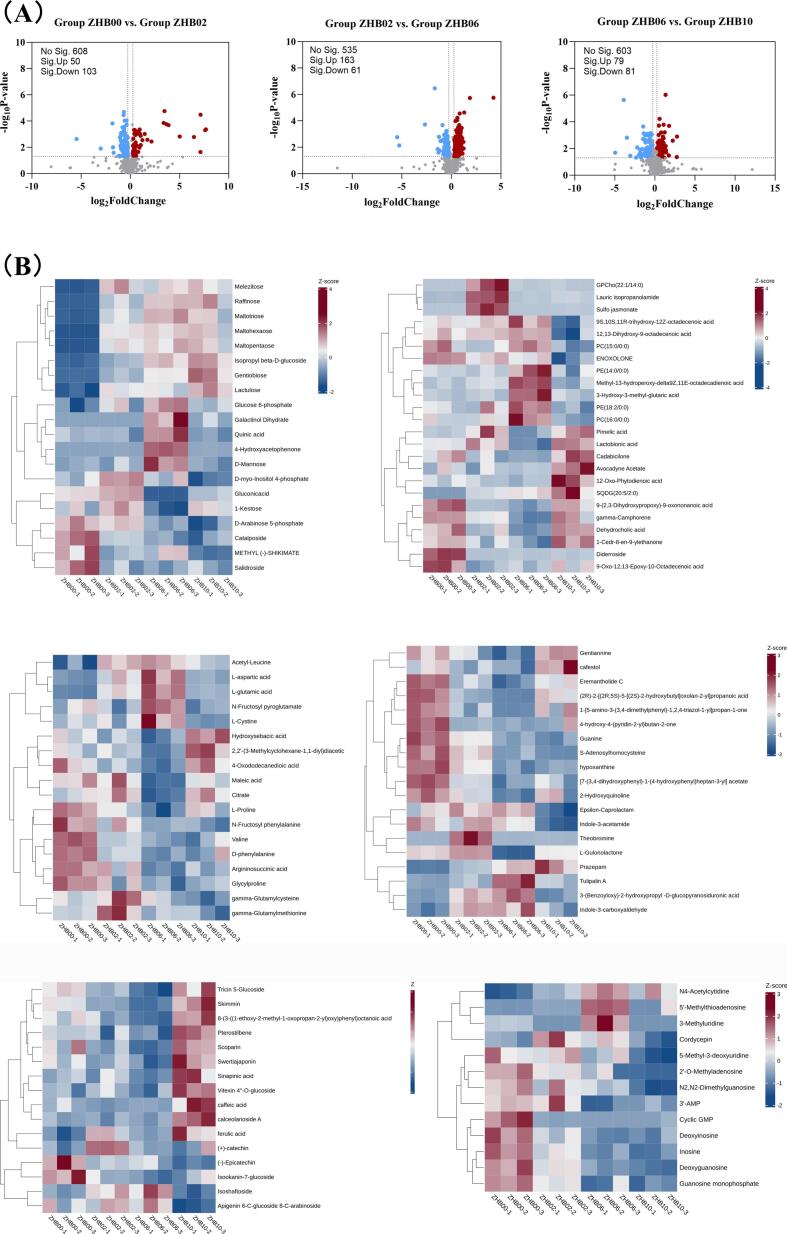

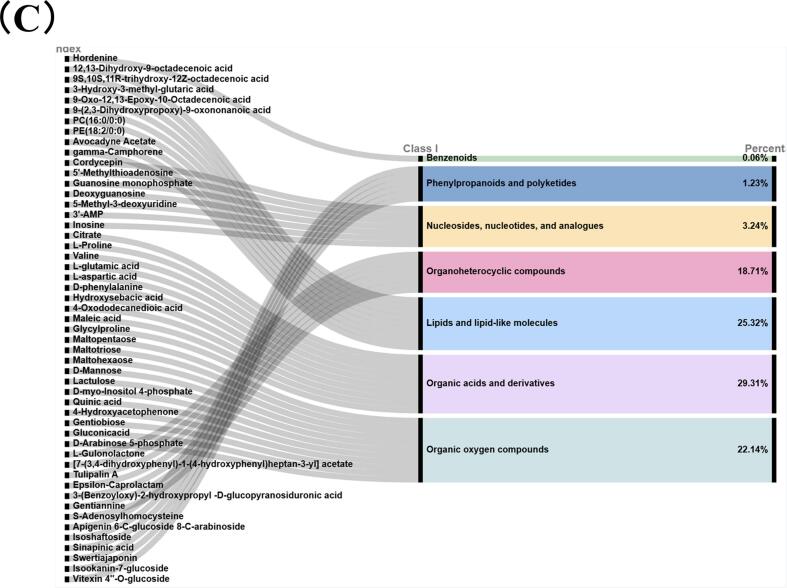
[cid:76f84e88$3$19d6325db43$Coremail$dengxiaoqing0$163.com]. [cid:4938c875$4$19d6325db43$Coremail$dengxiaoqing0$163.com].

A thorough analysis of metabolic patterns during fermentation was conducted by examining differential metabolites in each sample, which were visualized using volcano plots (Fig. 3A). Differential marker metabolites were identified using cutoff thresholds of fold change >1.2, variable importance in projection (VIP) > 1, and *P* < 0.05. Comparing the groups revealed dynamic shifts in metabolite expression profiles linked to varying BGS supplementation levels. Compared with those in ZHB00, 761, 759, and 763 metabolites were significantly altered in ZHB02, ZHB06, and ZHB10, respectively; among these, 28, 104, and 49 metabolites showed increased abundance, whereas 48, 26, and 55 showed decreased abundance. These results suggested that enzymatic hydrolysis promoted catabolic and anabolic activities in hull-less barley dough.

Based on the criteria of VIP > 1 and *P* < 0.05, 118 key differential compounds were identified in ZHB samples (Fig. 3B). Hierarchical clustering revealed that the four samples formed two main branches, that is, ZHB00 and ZHB10. This separation highlights notable differences in the metabolic profiles between the ZHB00 and ZHB10 groups. The ZHB00 branch contained a single subclass, whereas the experimental group's branch was divided into two subclasses, indicating more pronounced metabolic variation between ZHB10 and ZHB02/ZHB06, with relatively minor differences observed between ZHB02 and ZHB06. Cluster analysis across all group replicates revealed high similarity among biological replicates within each treatment, aligning with PCA findings. Compared with that in ZHB00, numerous compounds showed increased abundance in ZHB02, ZHB06, and ZHB10, whereas others showed decreased abundance, suggesting that BGS treatment significantly influenced the fermentation characteristics of hull-less barley–wheat dough.

**Fig. 4:**
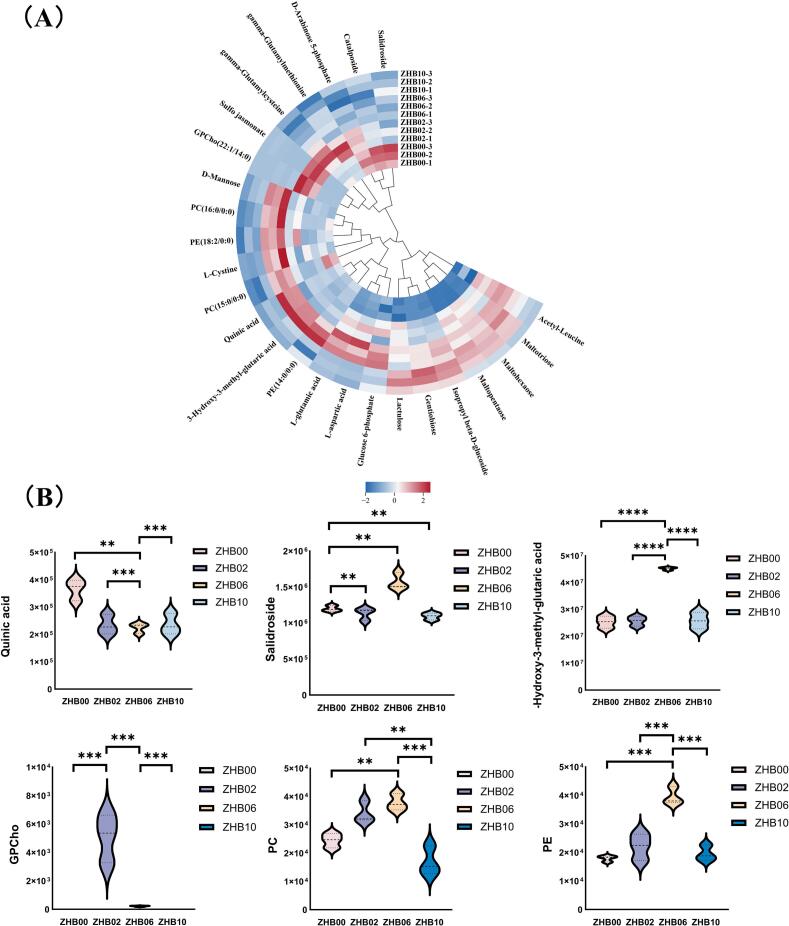

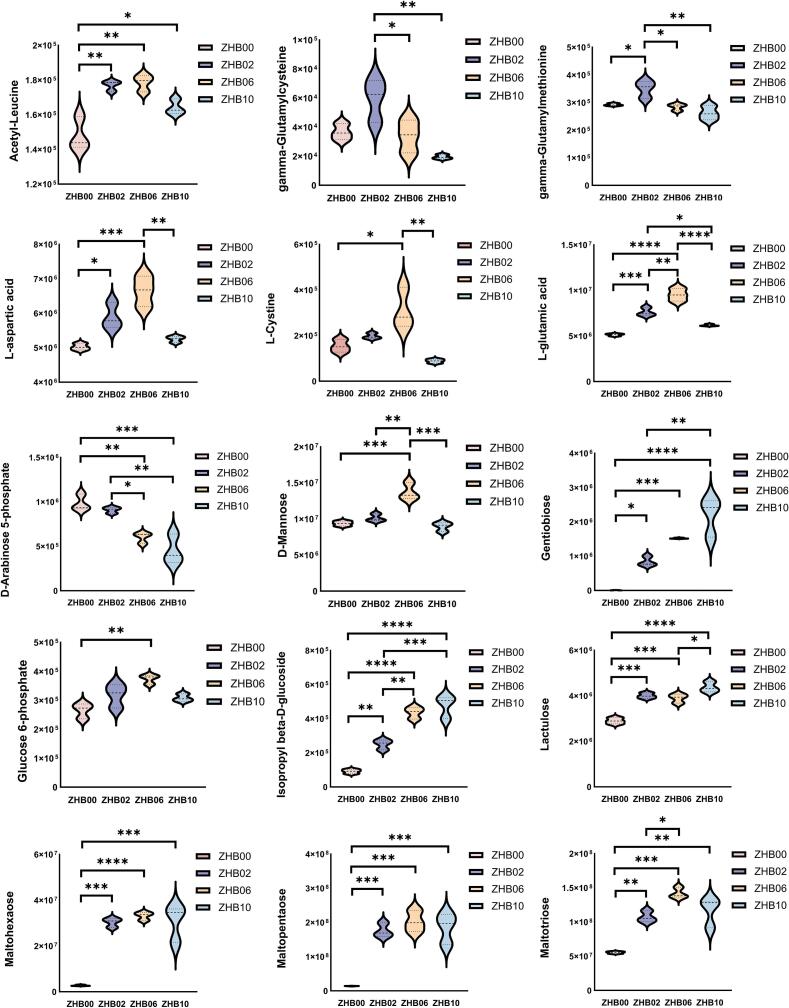
[cid:4cc45d66$5$19d6325db43$Coremail$dengxiaoqing0$163.com]. [cid:46990676$6$19d6325db43$Coremail$dengxiaoqing0$163.com].

The differential compounds were selected as key metabolites for cluster analysis (Fig. 3C). The abundances of key organic acids and derivatives, lipids and lipid-like molecules, organic oxygen compounds, and organoheterocyclic compounds in hull-less barley differed markedly from those in the raw materials (Fig. 4A, B). Organic acids, which function as intermediate products of plant photosynthesis and respiration, are prevalent in fermented foods and typically originate from microbial hydrolysis of carbohydrates. These compounds serve as substrates for the formation of various flavor compounds, including aldehydes, ketones, and esters (Abeijón Mukdsi, Maillard & Medina, 2018). In the current study, most detected organic acid–related compounds were amino acids, which not only contribute to protein synthesis but also play crucial roles in energy metabolism, stress responses, and metabolic regulation in plants.

Analysis revealed a range of flavor-contributing amino acids in the samples, including glutamic acid, aspartic acid, and tyrosine. These amino acids influence taste by participating in peptide formation and serving as precursors for the generation of small-molecule alcohols, esters, pyrazines, indoles, and hydrocarbons via deamination, decarboxylation, and Maillard reactions, thereby shaping the volatile flavor profile of food products (Hua et al., 2024). In the ZHB00 group, higher expression levels of argininosuccinic acid, glycylproline, *d*-phenylalanine, valyl-proline, and N-fructosyl phenylalanine were detected. The ZHB02 and ZHB06 groups exhibited significant increases in the relative concentrations of γ-glutamylcysteine, γ-glutamylmethionine, acetyl-leucine, L-aspartic acid, L-glutamic acid, and L-cystine.

Notably, the concentration of the bitter-tasting amino acid N-fructosyl phenylalanine was downregulated, whereas that of the umami amino acid glutamic acid was upregulated. These findings suggested that BGS-mediated enzymatic hydrolysis facilitated the release of umami amino acids from hull-less barley. These findings are consistent with those of Dilrukshi et al. (2022), who reported that extrusion processing significantly alters protein amino acid composition, potentially owing to protein structural unfolding and increased specific surface area. Hence, the enzymatic action of BGS may enhance protein accessibility and subsequent decomposition. The increased essential amino acid content suggests that BGS supplementation may improve the nutritional profile of hull-less barley–wheat whole grain dough. These amino acids and related compounds are incomplete degradation products of protein digestion or catabolism, many of which possess physiological activities.

It is postulated that the increased abundance of differential metabolites in lipids and lipid-like molecules predominantly results from the disruption of the viscous BG network. This degradation releases lipids, allowing them to participate in oxidative pathways, thereby facilitating the generation of flavor compounds. In the ZHB02 group, elevated levels of glycerophosphocholine (GPCho) and sulfojasmonate were observed, likely due to the relatively limited degradation of BG and the resulting high viscosity, which entraps lipid molecules, restricting their mobility and reactivity. The introduction of BGS led to the breakdown of glucan chains, disruption of the polymeric network, and subsequent release of bound free fatty acids and glycerides. With increasing BGS supplementation, significantly increased levels of 3-hydroxy-3-methyl-glutaric acid, phosphatidylethanolamine, and phosphatidylcholine (PC) were detected. Conversely, in the ZHB10 group, the abundance of glycerophospholipid declined markedly. The released glycerophospholipids may have redistributed to the air–liquid interfaces or formed complexes with starch and proteins. Furthermore, marked increases in avocadyne acetate, sulfoquinovosyl diacylglycerol (SQDG), 12-oxo-phytodienoic acid, and glycolipids were observed. Collectively, these results suggest that BGS modulates lipid hydrolysis and oxidation pathways by influencing substrate availability, thus impacting the metabolic activity of lactic acid bacteria and yeast. Moreover, lipid peroxidation can adversely affect food quality and may pose health concerns. The enzymatic degradation of BG by BGS into oligosaccharides provides an additional carbon source for yeast metabolic processes. Acids produced during yeast fermentation lower dough pH, potentially suppressing lipoxygenase activity, reducing polyunsaturated fatty acid oxidation, and thus diminishing lipid peroxide formation. This mechanism ultimately contributes to improved dough flavor profiles and enhanced quality stability.

Organic oxygen compounds comprise a substantial proportion of identified metabolites, with carbohydrates being the most prevalent. Carbohydrates play a vital role in plant cellular physiology, functioning as both structural components and metabolic substrates. Additionally, they are responsible for imparting the distinctive sweetness found in fermented foods. In the ZHB00 group, salidroside, catalposide, and D-arabinose 5-phosphate were present at relatively high levels. In contrast, the ZHB06 group exhibited higher levels of D-mannose, quinolinic acid, and glucose 6-phosphate. Upon its release, hexokinase phosphorylates D-mannose to form mannose-6-phosphate, which is converted by phosphomannose isomerase to fructose-6-phosphate before entering the glycolytic pathway or being transformed into glucose-6-phosphate via gluconeogenesis (Nakajima et al., 2018). Glucose-6-phosphate, a common intermediate in numerous sugar metabolism pathways and abundant in fruits and vegetables (Vanderwall & Gendron, 2023), decreased with increasing BGS supplementation. This decrease may be attributed to its breakdown via the hexose degradation and methylerythritol phosphate pathways (Plaxton, 1996). In the ZHB10 group, several organic oxygen compounds were significantly elevated, including gentiobiose, isopropyl β-D-glucoside, maltohexaose, maltopentaose, maltotriose, and lactulose. Gentiobiose is produced from *Aureobasidium pullulans* β-1,3/1,6-glucan under pH 4–6 conditions (Hirabayashi et al., 2017), consistent with our earlier analysis. Adding BGS degrades BG into low-molecular-weight products, liberating entrapped carbohydrates and potentially exposing substrates to endogenous enzymes, thereby accelerating their hydrolysis into disaccharides and monosaccharides (Streb et al., 2012). However, the observed reduction in certain monosaccharides, such as D-mannose, may be owing to yeast utilization of these sugars as additional carbon sources during fermentation, leading to increased gas production and reduced residual sugar levels.

#### 3.6.3 Metabolic pathway enrichment analysis

**Fig. 5:**
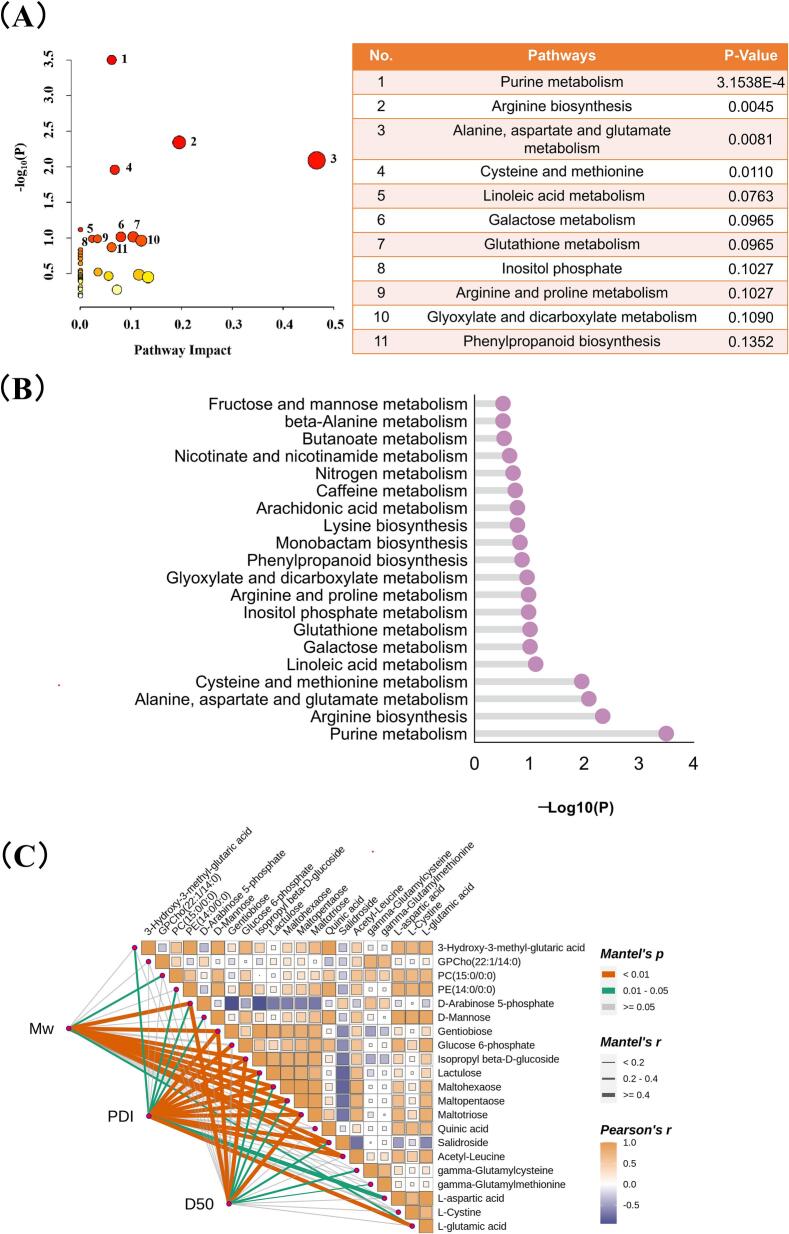
[cid:72218c23$7$19d6325db43$Coremail$dengxiaoqing0$163.com]

Relevant metabolites were mapped and annotated to KEGG pathways (Fig. 5 A), revealing significant enrichment in several metabolic pathways: purine metabolism; arginine biosynthesis; alanine, aspartate, and glutamate metabolism; cysteine and methionine metabolism; and linoleic acid metabolism (Fig. 5B). Linoleic acid metabolism is particularly important for shaping the aroma profiles of fermented foods. As a polyunsaturated fatty acid that humans cannot synthesize, linoleic acid is primarily metabolized within the phospholipid bilayer of cell membranes. This process is initiated by the dehydrogenase-catalyzed oxidation of linoleic acid into linolenic acid, which is further oxidized to eicosapentaenoic acid. The subsequent decarboxylation yields arachidonic acid. Linoleic acid metabolism supports critical physiological functions. Arachidonic acid—produced during this process—is essential for maintaining cell membrane structure and function and for supporting cardiovascular development, vision, and cognitive functions (Rayaroth et al., 2021). Linoleic acid also serves as a key precursor for flavor in fermented foods. Through the lipoxygenase pathway, it is converted to hexanal and its isomers, which are reduced by alcohol dehydrogenases into their respective alcohols. These alcohols are esterified by alcohol acyltransferases into hexyl esters, contributing to fruity and floral aroma notes. Oxidized linoleic acid products, such as hexanal and 2,4-decadienal, impart grassy and fatty notes, enhancing the overall flavor profile.

Oligosaccharides such as maltotriose and gentiobiose, formed from BGS hydrolysis, serve as carbon sources and participate in the glycolytic pathway in yeast. When BG is degraded by BGS, it produces low-molecular-weight sugars such as glucose, which is phosphorylated at the C6 position by hexokinase or glucokinase, creating glucose-6-phosphate—a key molecule linking glycolysis, glycogen synthesis, and the pentose phosphate pathway. Additionally, this process may contribute to the formation of nucleoside-related compounds through the Maillard reaction.

Purines and pyrimidines are essential nucleosides, nucleotides, and nucleic acids. In cereal grains, these bases exist as DNA and RNA within nuclei and mitochondria. During the fermentation of hull-less barley–wheat dough, yeast releases nucleases that break down nucleic acids from barley and wheat, resulting in purine nucleotide release. These nucleotides are further degraded into purine bases and partially converted into uric acid or urea. Yeast can also synthesize purine nucleotides de novo from substrates such as glycine and formate to support their proliferation. Pyrimidine metabolism follows a similar pathway during fermentation, in which yeast degrades nucleotides from cereal nucleic acids into pyrimidine bases, which are then further metabolized to β-alanine or β-aminoisobutyrate. These metabolites eventually enter the tricarboxylic acid cycle or are excreted (Cai et al., 2018). Notably, β-alanine contributes to the subtle umami or sweet aftertaste of steamed bread.

Hull-less barley is a cereal characterized by high protein content, with amino acid metabolism a particularly significant metabolic pathway. BG, proteins, and starch interact to form a three-dimensional network structure (Li et al., 2023). The structural breakdown of BG accelerates protein hydrolysis, a key biochemical process in flavor development. The released free amino acids are subsequently catabolized into keto acids, amines, and sulfur-containing compounds (Ter et al., 2024). During fermentation, yeast produces alcohols, free fatty acids, and free amino acids, thereby imparting umami and sweet flavors to the final fermented products. Compared with that of wheat, the amino acid profile of hull-less barley is more balanced, with substantially higher levels of aspartic acid and glutamic acid. The metabolism of alanine, aspartate, and glutamate is closely linked to the protein composition of raw materials, microbial activity, and fermentation conditions. These three amino acids serve not only as precursors for protein synthesis but as key contributors to flavor formation, energy metabolism, and the development of functional components (Chen et al., 2025).

Alanine primarily arises from protein degradation and yeast synthesis. Yeast secretes proteases that hydrolyze gluten proteins, thereby facilitating the release of alanine. Alanine is also produced by yeast through transamination reactions involving glycolytic intermediates such as pyruvate and glutamate. Pyruvate generated in this process predominantly participates in the glucose–pyruvate and tricarboxylic acid cycle. Alanine contributes to the mildly sweet taste and improved mouthfeel of hull-less barley steamed bread, while also aiding in nitrogen balance and dough expansion.

Aspartate is primarily derived from globulins in hull-less barley and enzymatically hydrolyzed wheat proteins. However, it can also be synthesized from oxaloacetate via transamination. Aspartate provides the amino groups necessary for urea synthesis, thereby reducing ammonia accumulation. Furthermore, it can be converted to asparagine, which possesses excellent antioxidant properties. By lowering free ammonia levels during fermentation, aspartate decreases undesirable off-flavors and supports the production of antioxidative asparagine.

Glutamate primarily originates from wheat gluten and is released via yeast activity. It can also be synthesized from α-ketoglutarate via glutamate dehydrogenase, in the presence of ammonia. Free glutamate is a key contributor to the umami taste of steamed bread, enhancing the nutty aroma characteristic of hull-less barley and improving the overall flavor profile (Xun et al., 2024).

### 3.7 Correlation between BG structural parameters and fermentation metabolites

The Mantel test enables comparisons between distance matrices, providing insights into relationships among variables (Fig. 5C). Analysis revealed strong correlations between metabolites and BG structure. Overall, high Mw was inversely associated with most carbohydrate metabolites but showed a positive association with certain phospholipids. D_50_ demonstrated a clear negative correlation with amino acids and their derivatives. These results suggest that BGS treatment influences carbohydrate, lipid, and amino acid metabolic pathways by altering the physicochemical properties of BG.

Specifically, Mw showed strong negative correlations with oligosaccharides, including maltotriose, maltohexaose, gentiobiose, and lactulose. This confirmed the direct degradative action of BGS on BG, with these oligosaccharides serving as direct enzymatic products and carbon sources for microorganisms. Conversely, Mw correlated positively with GPCho and PC, indicating that high-molecular-weight BG might influence microbial community structure. The negative associations between D_50_ and amino acids and their derivatives, including L-glutamate, l-aspartate, γ-glutamylcysteine, and acetyl-leucine, suggest that BG psize significantly impacts amino acid metabolism during fermentation. Hence, BGS-induced reduction in BG psize likely accelerates protein hydrolysis and amino acid metabolism by increasing substrate accessibility.

PDI exhibited more intricate correlation patterns, including associations with organic acid metabolites such as quinic acid and 3-hydroxy-3-methylglutaric acid. Given that PDI reflects the homogeneity of BG molecular sizes, a higher PDI may provide a broader spectrum of carbon sources for microbial communities, stimulating a wider range of metabolic pathways that encompass those associated with phenolic acids and intermediates in branched-chain amino acid metabolism.

## 4 Conclusion

This study elucidates the mechanisms by which BGS modulates the BG network and alters the metabolic profile in hull-less barley dough. BGS hydrolyzes glycosidic bonds in BG, thereby decreasing Mw, psize, and crystallinity. This structural modification disrupts the hydrogen-bonding network in the dough matrix, thereby reducing system viscosity and enhancing processing performance. Furthermore, BGS treatment improves molecular mobility and releases encapsulated components such as starch and proteins, increasing substrate accessibility for microbial utilization and enzymatic activity, thereby reshaping the metabolic landscape. Metabolomic profiling identified 768 metabolites, with BGS treatment notably increasing the levels of organic acids and their derivatives, lipids and lipid-like molecules, and organic oxygen compounds. Integration of structural and metabolic data revealed that BG degradation facilitates the release of flavor precursors, including glutamate and aspartate, enhances aroma formation via lipid oxidation pathways, and enhances yeast fermentation by increasing the availability of carbon sources.

Thus, BGS serves as an effective enzymatic modifier, with the potential to address processing constraints associated with high BG content in hull-less barley and improve both technological attributes and metabolic quality. This study establishes a theoretical basis for enhancing the quality of hull-less barley-based products. Future studies should investigate the formation kinetics of key metabolites under BGS treatment and their potential interactions with gut microbiota to support its utilization in health-oriented food applications.

## CRediT authorship contribution statement

**Ziyuan Guo:** Writing – original draft, Formal analysis. **Xin Wang:** Writing – original draft, Formal analysis. **Xiaoqing Deng:** Project administration, Methodology, Funding acquisition. **Luyu Fan:** Writing – review & editing, Software. **Qun Shen:** Validation, Data curation. **Xiang Li:** Supervision, Resources, Investigation. **Xiaomeng Wu:** Supervision, Resources, Investigation.

## Declaration of competing interest

The authors declare that they have no known competing financial interests or personal relationships that could have appeared to influence the work reported in this paper.

## Data Availability

Data will be made available on request.
